# A Phenotypic High-Throughput Screen Identifies Small Molecule Modulators of Endogenous RGS10 in BV-2 Cells

**DOI:** 10.1021/acs.jmedchem.4c01738

**Published:** 2024-11-15

**Authors:** Shwetal Talele, Stephanie Gonzalez, Julia Trudeau, Ahmad Junaid, Cody A Loy, Ryan A. Altman, Benita Sjögren

**Affiliations:** † Department of Pharmaceutical Sciences, University of California, Irvine, Irvine, California 92697, United States; ‡ Borch Department of Medicinal Chemistry and Molecular Pharmacology, Purdue University, West Lafayette, Indiana 47907, United States

## Abstract

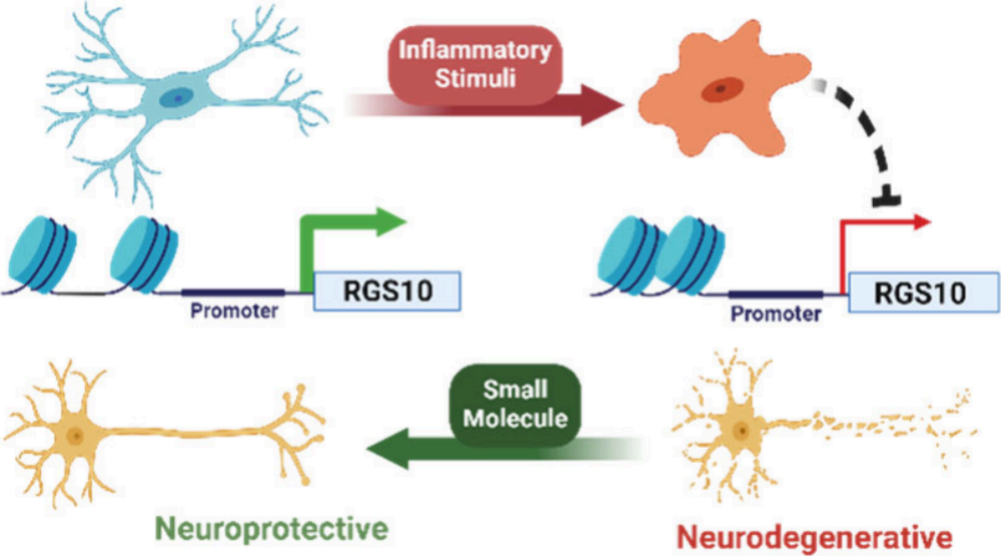

Chronic dysregulation of microglial phenotypic balance contributes to prolonged neuroinflammation and neurotoxicity, which is a hallmark of neurodegenerative diseases. Thus, targeting microglial inflammatory signaling represents a promising therapeutic strategy for neurodegenerative diseases. Regulator of G protein Signaling 10 (RGS10) is highly expressed in microglia, where it suppresses pro-inflammatory signaling. However, RGS10 is silenced following microglial activation, augmenting inflammatory responses. While modulating RGS10 expression is a promising strategy to suppress pro-inflammatory microglial activation, no chemical tools with this ability exist. We developed a phenotypic high-throughput assay to screen for compounds with the ability to reverse interferon-γ (IFNγ)-induced RGS10 silencing in BV-2 cells. Identified hits had no effect on RGS10 expression in the absence of stimulus or in response to lipopolysaccharide (LPS). Furthermore, the hits reversed some of the inflammatory gene expression induced by IFNγ. This is the first demonstration of the potential for small molecule intervention to modulate the RGS10 expression in microglia.

## Introduction

Microglia, the resident macrophages of the central nervous system (CNS), are the primary drivers of chronic neuroinflammation, a hallmark of several age-related neurodegenerative diseases (NDs) including Alzheimer’s Disease (AD) and Parkinson’s Disease (PD).^1−4^ Microglia exist across a broad spectrum of phenotypes depending on specific stimuli and the resulting signaling pathways that are activated. In a healthy brain, resting/ramified microglia exhibit highly branched processes that actively survey, detect, and respond to environmental signals of infection or damage.^5^ This is achieved by the expression of diverse receptors, including toll-like receptors (TLRs), which are the target of the bacterial endotoxin lipopolysaccharide (LPS) and other pathogen-associated ligands,^6^ as well as receptors for interferon-γ (IFNγ), a central immune mediator of immune cell crosstalk that amplifies inflammatory signaling, and purinergic G protein-coupled receptors (GPCRs; P2YRs), which respond to nucleotides released from neighboring dying cells. Signaling by all three receptor types triggers morphological and functional transformations to various activated states,^7−9^ accompanied by functional responses, such as migration, phagocytosis,^10^ and release of inflammatory mediators, that contribute to either a reparative or a neurotoxic response.^11,12^ Chronic dysregulation of the microglial phenotypic balance toward pro-inflammatory phenotypes contributes to prolonged neuroinflammation and neurotoxicity, which promotes disease progression in age-related NDs.^13^ Therefore, targeting microglial inflammatory signaling serves as a promising therapeutic strategy to improve clinical prognosis for inflammatory NDs for which effective corrective therapies are lacking.

GPCRs are broadly involved in (patho)physiological functions, including strong implications in multiple NDs.^14^ GPCR agonists mediate their effects by promoting the exchange of GDP for GTP on a Gα subunit of heterotrimeric G proteins^15,16^ and activation of downstream effectors. Signal deactivation by GTP hydrolysis to GDP is accelerated by GTPase activating proteins (GAPs),^17^ most notably the Regulator of G protein Signaling (RGS) protein superfamily.^18−21^ In the R12 subfamily of RGS proteins, RGS10 has been proposed to serve a key anti-inflammatory and neuroprotective role in microglia.^22^ RGS10 is a selective GAP at activated Gα_i_ proteins, thereby negatively regulating signaling through Gi-coupled GPCRs,^23^ including many chemokine receptors. RGS10 is highly expressed in microglia, where it suppresses pro-inflammatory signaling and protects against inflammation-induced neurotoxicity.^22^ RGS10^–/–^ mice exhibit increased microglia activation, and primary microglia isolated from these mice display dysregulated signaling, including enhanced production of pro-inflammatory cytokines (particularly TNFα and COX-2),^24^ interleukins, and prostaglandins.^25,26^ RGS10^–/–^ mice are also more susceptible to dopaminergic neuron loss in the substantia nigra pars compacta (SNpc) than wildtype mice.^26^ Moreover, adenovirus-mediated RGS10 overexpression in the SNpc of rats attenuates microgliosis and protects against 6-OHDA-induced degeneration of dopaminergic neurons.^27^

The anti-inflammatory role of microglial RGS10 in vivo has been consistently modeled in microglial cell lines, most extensively in the BV-2 mouse microglial cell model.^28^ RGS10 knock-down or knockout in BV-2 cells enhances inflammatory gene expression triggered by LPS.^26,27^ Similarly, RGS10 overexpression suppresses pro-inflammatory cytokine release and neurotoxicity.^26,27^ Taken together, the ability to simultaneously suppress microglial pro-inflammatory gene expression and neurotoxicity while promoting neuroprotective functions makes RGS10 an attractive target for development of therapeutics for neuroinflammatory diseases. However, the mechanisms and pathway specificity by which RGS10 acts are undefined, hampering the development of RGS10-targeted therapies. Critically, the extent to which the effects of RGS10 are mediated by its canonical GAP activity remains unknown. As an example, LPS mainly acts through non-GPCR, Toll-like receptors (TLRs), and the ability of RGS10 to regulate LPS-stimulated inflammatory gene expression is G protein-independent.^24^

RGS10 is transcriptionally silenced in vivo by endogenous inflammatory signals,^29^ and LPS or TNFα reduces RGS10 expression levels by up to 80% in both primary microglia and BV-2 cells.^26,29^ Furthermore, direct suppression of RGS10 expression by 50–80% using siRNA induces strong upregulation of inflammatory gene expression, indicating that the suppression of RGS10 triggered by receptor activation is sufficient to significantly increase pro-inflammatory signaling. Together, these data suggest that silencing of RGS10 by inflammatory stimulation amplifies pro-inflammatory microglial signaling that contributes to chronic neuroinflammation. Identifying compounds that can reverse RGS10 silencing in activated microglia could therefore serve as promising leads for suppressing neuroinflammation in the treatment of NDs. In addition, these compounds would also serve as useful tools to elucidate mechanisms underlying the role of RGS10 in microglial inflammatory signaling, of which the majority are unknown. To this end, we developed and employed an unbiased high-throughput screening strategy to identify small molecules with the ability to reverse IFNγ-induced RGS10 silencing in BV-2 cells. We identified a series of compounds that reverse IFNγ-induced but not LPS-induced RGS10 silencing and display promising effects on IFNγ-induced inflammatory gene expression.

## Results

### RGS10 Expression Is Suppressed in Response to Inflammatory Stimuli in BV-2 Cells

**Figure 1:**
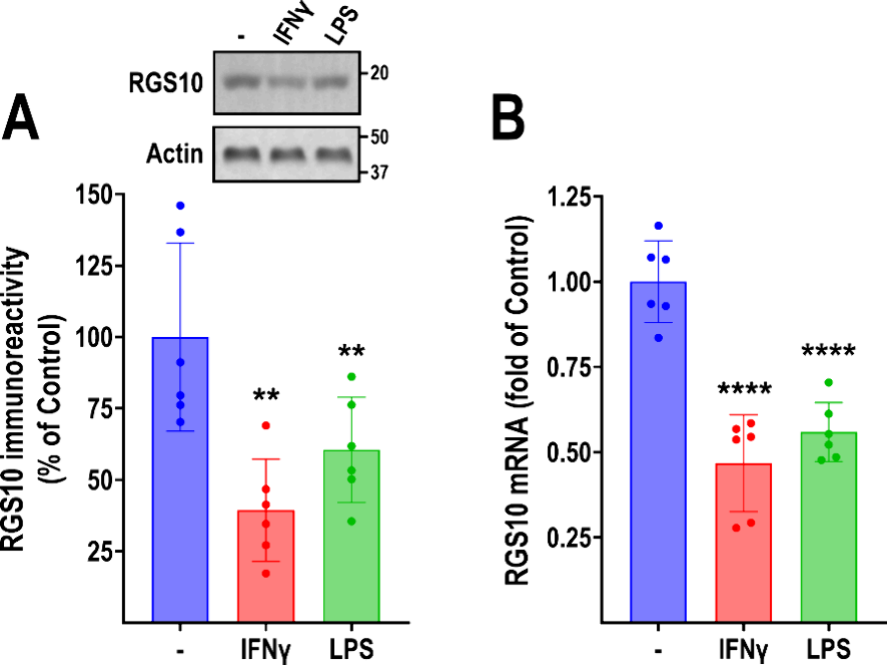
RGS10 is suppressed in BV-2 cells in response to inflammatory stimuli. BV-2 cells were treated with 10 ng/mL IFNγ or 10 ng/mL LPS (24 h) and subjected to Western blot (A) and qRT-PCR (B) to assess RGS10 protein and mRNA levels, respectively. IFNγ and LPS significantly reduced both the RGS10 protein and mRNA levels. Representative blot (A) and quantification of 6 independent experiments (A, B). **P < 0.01; ****P < 0.0001 using one-way ANOVA with Dunnet’s post hoc test for pairwise comparisons.

To identify small molecules that reverse the silencing of RGS10 expression in microglia, we utilized the murine BV-2 cell line. BV-2 cells are a validated stable cell line of microglial origin that expresses high levels of RGS10 protein and responds to inflammatory stimuli in a manner consistent with microglial activation. Previous studies demonstrating the neuroprotective role of RGS10 following microglial activation as well as the transcriptional silencing that occurs following microglial activation have almost exclusively utilized LPS as the inflammatory stimulus. Given that LPS is of bacterial origin and may not reflect the mode of microglial activation occurring in neurodegenerative diseases, we first aimed to demonstrate that RGS10 is silenced by other, endogenous triggers as well. BV-2 cells were treated with either IFNγ or LPS (both at 10 ng/mL) for 24 h and subjected to Western blotting and qRT-PCR. RGS10 protein levels were significantly reduced by both IFNγ and LPS (56% and 37%, respectively; Figure 1A). This reduction was mirrored at the mRNA level (61% by IFNγ and 39% by LPS; Figure 1B). These results show, for the first time, that RGS10 expression is silenced in BV-2 cells in response to IFNγ, and that this silencing is not unique to LPS.

### Development of a High-Throughput Assay That Detects Changes in Endogenous RGS10 Protein Levels

**Figure 2:**
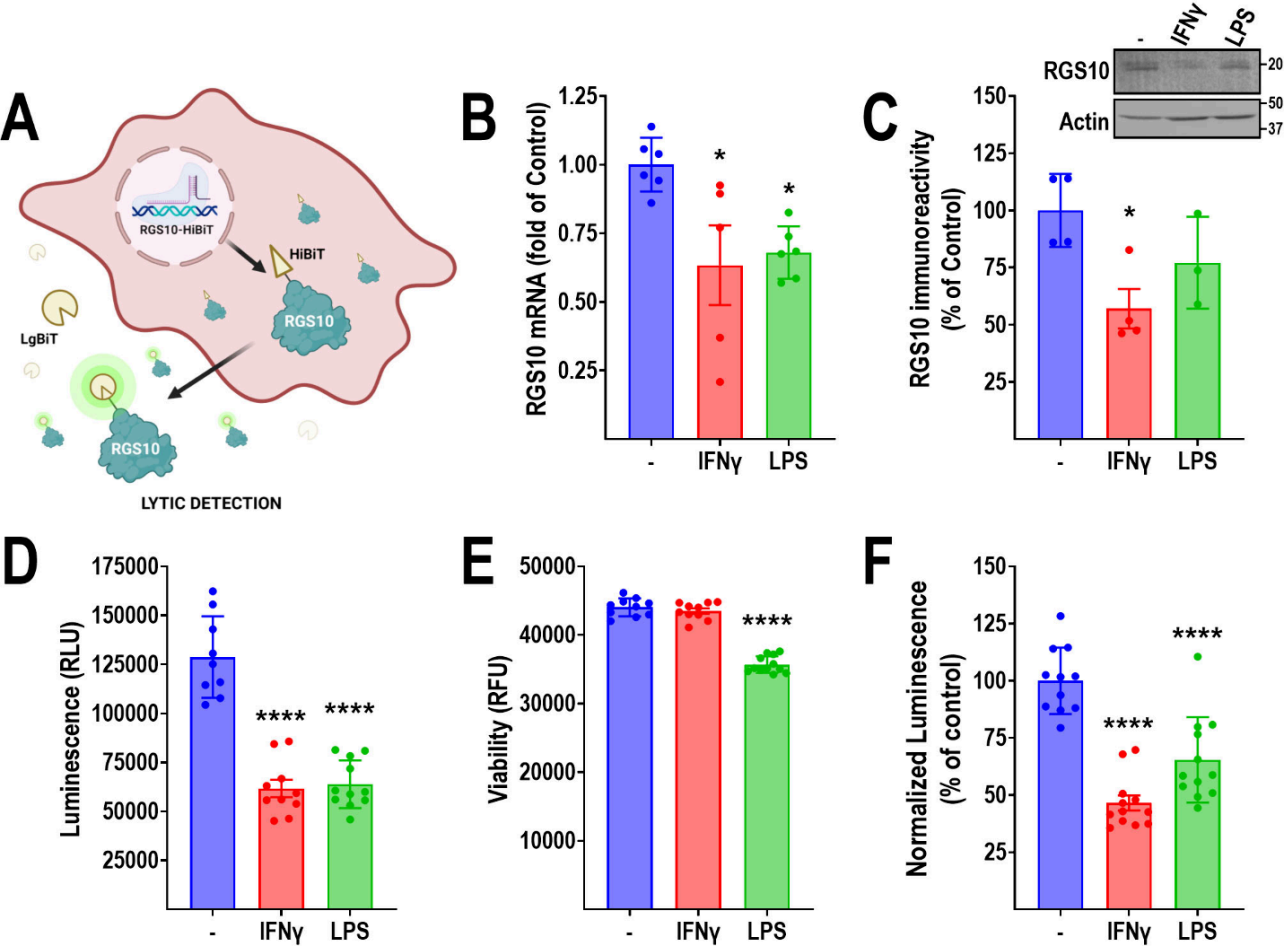
Development of a stable RGS10-HiBit cell line. A. Schematic of cell line and assay principle. The 11-residue HiBit tag was inserted using CRISPR/Cas9 at the C-terminus of endogenous RGS10 in BV-2 cells, enabling high-throughput detection of relative RGS10 protein levels using the Nano-Glo HiBit Lytic Detection Assay. Following single clone selection, the selected clonal cell line (BV-2-RGS10^HiBit^) was subjected to validation using (B) qRT-PCR and (C) Western blot. IFNγ significantly reduced the RGS10 mRNA and protein levels. The response to LPS was less robust, with only the reduction in mRNA reaching significance. D. IFNγ and LPS significantly reduced the BV-2-RGS10^HiBiT^ luminescence signal (RLU). E. LPS, but not IFNγ, significantly reduced the viability (RFU) of BV-2-RGS10^HiBiT^ cells. F. Normalized luminescence was obtained from D and E (RLU/RFU). *P < 0.05; ****P < 0.0001 using one-way ANOVA with Dunnet’s post hoc test for pairwise comparisons. Panel A was created with Biorender.

Small molecules with the ability to reverse RGS10 silencing in microglia would represent important probes to elucidate the neuroprotective roles of RGS10, and they are possible drug candidates for diseases associated with chronic neuroinflammation. Because the mechanisms involved in RGS10 silencing are largely unknown and because it involves genomic regions outside the RGS10 coding frame, we employed a mechanism-agnostic assay strategy to detect changes in endogenous RGS10 protein levels in BV-2 cells. NanoLuc Binary Technology (NanoBiT; Promega) is a split luciferase strategy, in which the LgBiT (17.6 kDa) subunit has little activity on its own but spontaneous, high affinity (∼1 nM) binding to an 11-amino-acid peptide (HiBiT) leads to enzyme complementation that restores NanoLuc Luciferase (Nluc) activity.^30,31^ We used CRISPR/Cas9 to insert HiBiT at the RGS10 C-terminus, enabling the high-throughput detection of changes in RGS10 protein levels under endogenous control of transcription and translation (Figure 2A). To reduce variability in the subsequent screen, we developed a stable single-clone cell line. Single clones were isolated by fluorescence-activated cell sorting (FACS) and, following expansion, tested for HiBiT luminescence signal in the absence or presence of IFNγ or LPS to identify a clone suitable for HTS. To ensure that the insertion of the HiBiT tag does not interfere with RGS10 expression, we validated that the final single-clone BV-2-RGS10^HiBiT^ cell line retained regulation of RGS10 mRNA and protein levels in response to IFNγ and LPS (Figures 2B, C). IFNγ induced robust silencing of both RGS10 mRNA and protein; however, the response to LPS was less pronounced and did not reach significance at the protein level. Our BV-2-RGS10^HiBiT^ cell line was optimized using the Nano-Glo HiBit Lytic Detection Assay, and both IFNγ and LPS (both at 10 ng/mL; 24 h) caused robust decreases in luminescence signal (Figure 2D). We simultaneously assessed cell viability using a cell permeable fluorogenic protease substrate (glycylphenylalanyl-aminofluoro-coumarin; GF-AFC),^32^ which can be multiplexed with a luminescent readout without interfering with the luciferase signal. We previously utilized this multiplexing in several screens,^33−35^ adding the benefit of identifying general compound toxicity at an early stage. LPS caused significant reductions in cell viability, whereas IFNγ did not (Figure 2E). When normalized to viability, LPS displayed a less robust suppression of RGS10^HiBiT^ luminescence than IFNγ (Figure 2F). Because IFNγ displayed a more robust suppression and less toxicity than LPS, and because LPS is of bacterial origin, we opted to use IFNγ as the inflammatory stimulus for our primary screening paradigm. We performed extensive assay optimization to ensure the maximum quality of the assay prior to screening. We optimized treatment conditions (time, temperature, and volume of reagents), cell density, buffer optimization and DMSO tolerance. While the luminescent signal was stable up to 60 min, the maximum signal occurred at 30 min. We also observed no effect on either viability or luminescence signal at DMSO concentrations <1%. Our final assay demonstrated robust quality, as measured using the Z factor >0.5,^36^ as determined by comparing the response in the absence and presence of IFNγ.

### HTS to Identify Small Molecule RGS10 Modulators

**Figure 3:**
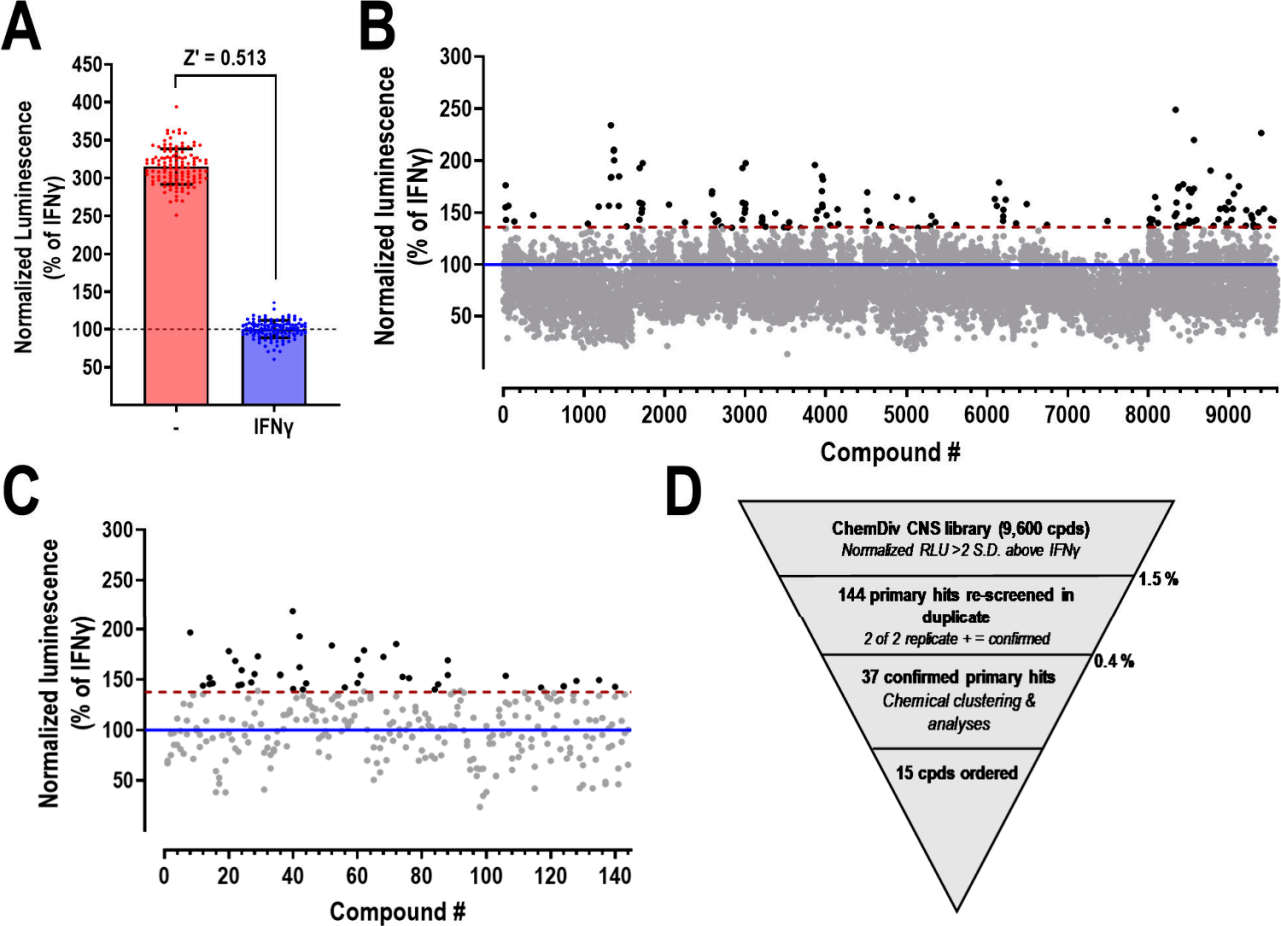
Primary screen for small molecule RGS10 modulators. A. Representation of the Z-factor obtained in the primary screen, as determined in the absence (−) and presence (IFNγ) of IFNγ (10 ng/mL; 48 h). *N* = 128 for each condition. B. Scatterplot of primary screen. 9,600 compounds from the ChemDiv CNS BBB library. C. Scatter plot for hit confirmation. Data presented as normalized luminescence (RLU/RFU) and expressed as % of IFNγ alone. Blue line represents average response in the presence of IFNγ (10 ng/mL; 48 h); Red dotted line represents 2 SD above that of IFNγ alone; Hits in A and B are highlighted in black. D. Screening funnel with hit rates for the primary screen and hit confirmation.

Small molecules with the ability to reverse RGS10 silencing that occurs upon inflammatory stimuli would be useful early probes to study the effects of RGS10 on microglial activation. Therefore, our primary screen was designed to identify compounds that would reverse IFNγ-induced (10 ng/mL; 48 h) RGS10 silencing. 9,600 compounds from the ChemDiv CNS BBB collection were screened in this paradigm. This library is designed for targets relevant to CNS diseases, such as Alzheimer’s Disease and Parkinson’s Disease. Furthermore, the physical and chemical properties of compounds within this library are composed of structures that can effectively cross the blood–brain barrier (BBB), based on previously published prediction algorithms.^37,38^ Compounds were screened at 20 μM with IFNγ (10 ng/mL) added simultaneously. Hits were defined as compounds that increased RGS10 HiBit signal >2 SD above that obtained with IFNγ alone. The overall Z′ in the primary screen (comparing normalized luminescence ±IFNγ) was 0.513 (Figure 3A). The primary screen yielded 144 primary hits (1.5% hit rate; Figure 3B), which were subjected to hit confirmation in triplicate. This confirmation resulted in 37 confirmed hits and a final hit rate of 0.4% (Figure 3C; Table S2). The outline of the screen is schematically presented in Figure 3D.

We next performed chemical clustering of our hits as well as filtering out compounds with unfavorable properties. The analysis, as described in Materials and Methods, culminated in the identification of a total of 19 distinct clusters. The clustering strain was quantified to be 1.181, with a minimum threshold set at 1.0. The strain value serves as an indicator of clustering accuracy, for which a lower strain value suggests a more precise ordering within the clusters.^39^ Tanimoto similarity scores, used to assess the structural resemblance among the clusters, are detailed in Table S2. Dendrogram and distance matrix of the generated clusters are shown in Figures S1–S3. Following the clustering process, further detailed medicinal chemistry analysis addressed the clusters with multiple structures, enabling the prioritization of compounds based on favorable characteristics and their drug-like properties. For clusters bearing multiple compounds, derivatives were deprioritized based on the presence of labile substructures and oxidatively and metabolically sensitive moieties (e.g., anilines and/or S-containing moieties). This process narrowed down the number of hits to 15 that were chosen for further follow-up studies.

### Hit Validation

**Figure 4:**
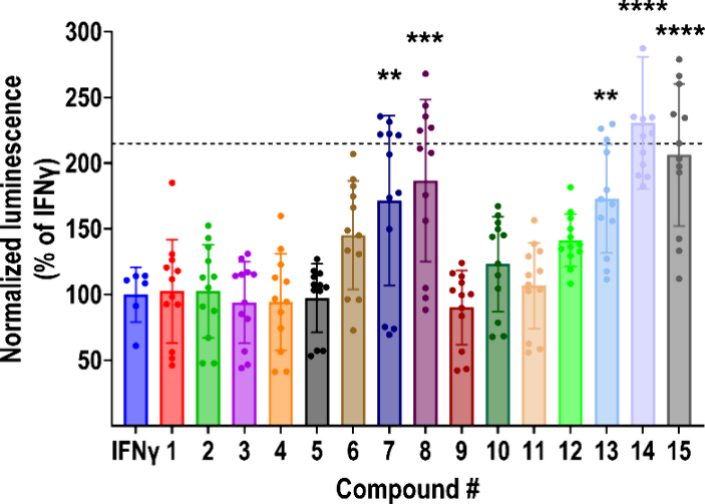
Validation of primary hits. 15 hits from the primary screen were assayed for their ability to reverse IFNγ-induced (10 ng/mL; 24 h) suppression of RGS10^HiBiT^ luminescence. Luminescence signal was normalized to viability (RLU/RFU) and expressed as % of the signal obtained with IFNγ alone. Dashed line represents the average signal in the absence of a stimulus. Five compounds, designated **7**, **8**, **13**, **14**, and **15**, significantly reversed IFNγ-induced suppression of RGS10^HiBiT^ luminescence. Results from four independent experiments run in triplicate. **P < 0.01; ***P < 0.001; ****P < 0.0001 using one-way ANOVA with Dunnet’s post hoc test for pairwise comparisons.

The 15 chosen hits from our primary screen were reordered from ChemDiv and subjected to the same assay paradigm as in the primary screen. Our primary screen was run with 48 h treatments to reduce variability and enhance IFNγ-induced RGS10 silencing. However, in all our follow-up studies, we reduced the treatment to 24 h to better reflect acute microglial activation and reduce off-target effects. Five of the compounds, designated **7**, **8**, **13**, **14**, and **15**, significantly reversed IFNγ-induced (10 ng/mL) suppression of RGS10 protein levels in this paradigm (Figure 4). Interestingly, none of the compounds reversed LPS-induced (10 ng/mL) RGS10 suppression, nor did they increase RGS10 levels in the absence of stimuli (Table 1), indicating that they act on a target along the IFNγ signaling axis. Additionally, computational PAINS assessment of these five structures did not reveal any alerts.^40,41^

**Table 1:** Summary of Single Point Confirmation of 15 Re-ordered Confirmed Hits; Effects of Compounds on RGS10 HiBit Signal Was Assessed under Basal Conditions or in the Presence of IFNγ or LPS (Both 10 ng/mL; 24 h)^a^

No.	CGF ID	ChemDiv ID	% IFN response	% LPS response	% Nonstimulated
**1**	CGF-0185111	M788-4605	102.4 ± 39.3	87.4 ± 11.8	104.8 ± 16.4
**2**	CGF-0185364	P194-2174	102.4 ± 35.2	105.8 ± 18.2	116.1 ± 22.8
**3**	CGF-0187664	S324-0173	94.0 ± 31.2	89.5 ± 15.6	97.1 ± 24.1
**4**	CGF-0188140	S342-0449	94.3 ± 36.7	90.3 ± 11.9	78.9 ± 13.5^a^^b^
**5**	CGF-0188561	S343-0670	97.4 ± 26.0	104.7 ± 14.7	85.6 ± 10.5
**6**	CGF-0188681	S348-2010	145.1 ± 41.3	113.8 ± 20.5	95.9 ± 24.6
**7**	CGF-0188707	S350-0115	171.6 ± 64.8 **	84.1 ± 6.9	69.6 ± 13.6 ***^b^
**8**	CGF-0188747	S350-0116	186.7 ± 61.6 ***	80.4 ± 18.6	56.8 ± 7.8 ***^b^
**9**	CGF-0188926	S348-1665	90.1 ± 28.2	N/A	156.8 ± 19.5 ****
**10**	CGF-0189561	S368-0654	123.2 ± 36.2	105.3 ± 23.4	113.8 ± 13.2
**11**	CGF-0190549	S425-0152	106.7 ± 32.7	121.7 ± 12.3	101.2 ± 8.3
**12**	CGF-0193338	C598-0583	141.3 ± 19.9	97.4 ± 17.2	104.6 ± 12.3
**13**	CGF-0185111	C522-3730	172.8 ± 41.4 **	116.9 ± 12.1	101.8 ± 17.8
**14**	CGF-0193870	F326-0563	230.6 ± 50.4 ****	92.8 ± 13.0	98.5 ± 23.9
**15**	CGF-0194281	L923–0739	206.2 ± 54.1 ****	104.1 ± 11.3	76.8 ± 28.3

a *P < 0.05; **P < 0.01; ***P < 0.001; ****P < 0.0001 using one-way ANOVA with Dunnet’s post hoc test for pairwise comparisons.

b HiBit signal *decreased* compared to control.

**Figure 5:**
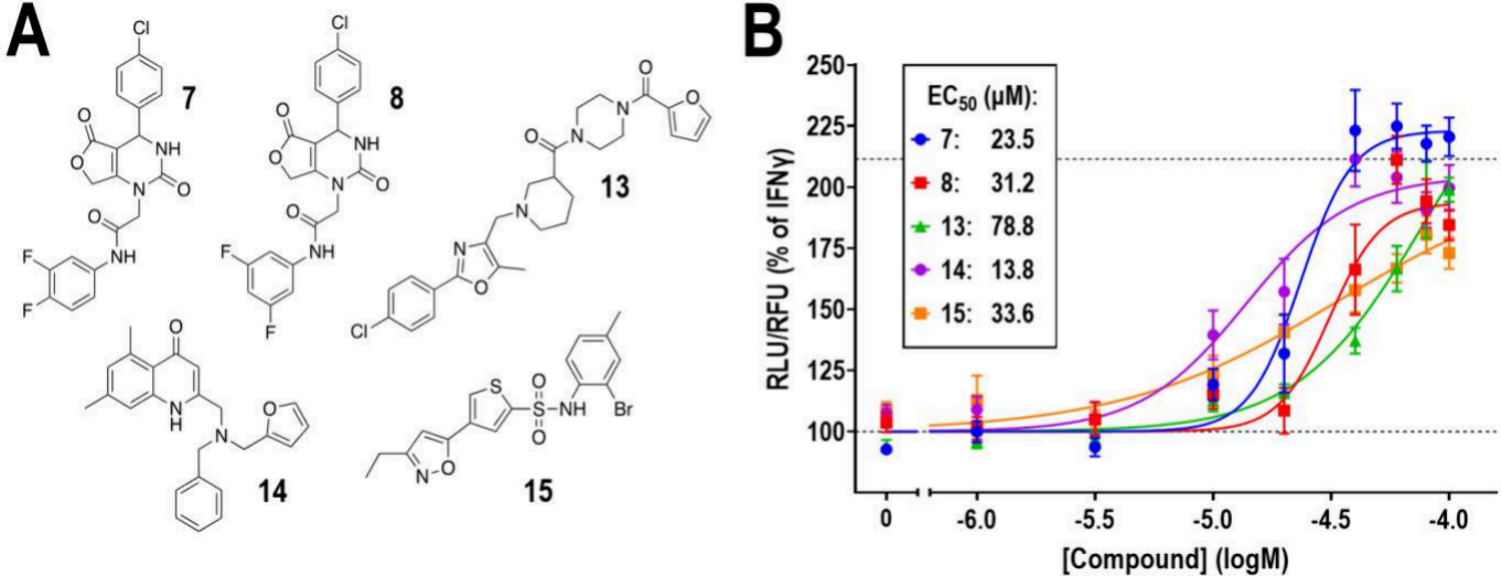
Dose–response and structures of validated hits. A. Structures of the five validated hits. Further information about clustering is depicted in Table S2. B. The five validated hits display concentration-dependent reversal of IFNγ-induced (10 ng/mL; 24 h) suppression of RGS10^HiBiT^ luminescence, with EC_50_ values displayed in the box. Concentration range 1–100 μM. Luminescence signal was normalized to viability (RLU/RFU) and expressed as % of the signal obtained with IFNγ alone. Dashed line represents the average signal in the absence of stimulus. All compounds display adequate Hill slopes (0.5–2). Compounds **7**, **8**, and **14** reach a maximum efficacy close to the response in the absence of a stimulus. Compounds **13** and **15** did not reach a maximum efficacy at the maximum concentration used. Results from 4 independent experiments run in triplicate.

Compounds **7**, **8**, **13**, **14**, and **15** (Figure 5A) were further assayed using a dose–response paradigm in the presence of IFNγ (10 ng/mL; 24 h). The concentration range in these assays was 1–100 μM. Due to solubility restrictions, we could not increase the maximum concentration above 100 μM. All five compounds increased RGS10 levels in a concentration-dependent manner, with EC_50_ values ranging from 13.8 to 78.8 μM (Figure 5B); however, the EC_50_ values for compounds **13** and **15** are approximate, as these did not reach maximum efficacy at 100 μM. Nevertheless, all five compounds reversed IFNγ-induced (10 ng/mL; 24 h) suppression of RGS10^HiBiT^ luminescence, with efficacies at, or close to, full reversal.

### Identified Hits Regulate Native RGS10 Levels

**Figure 6:**
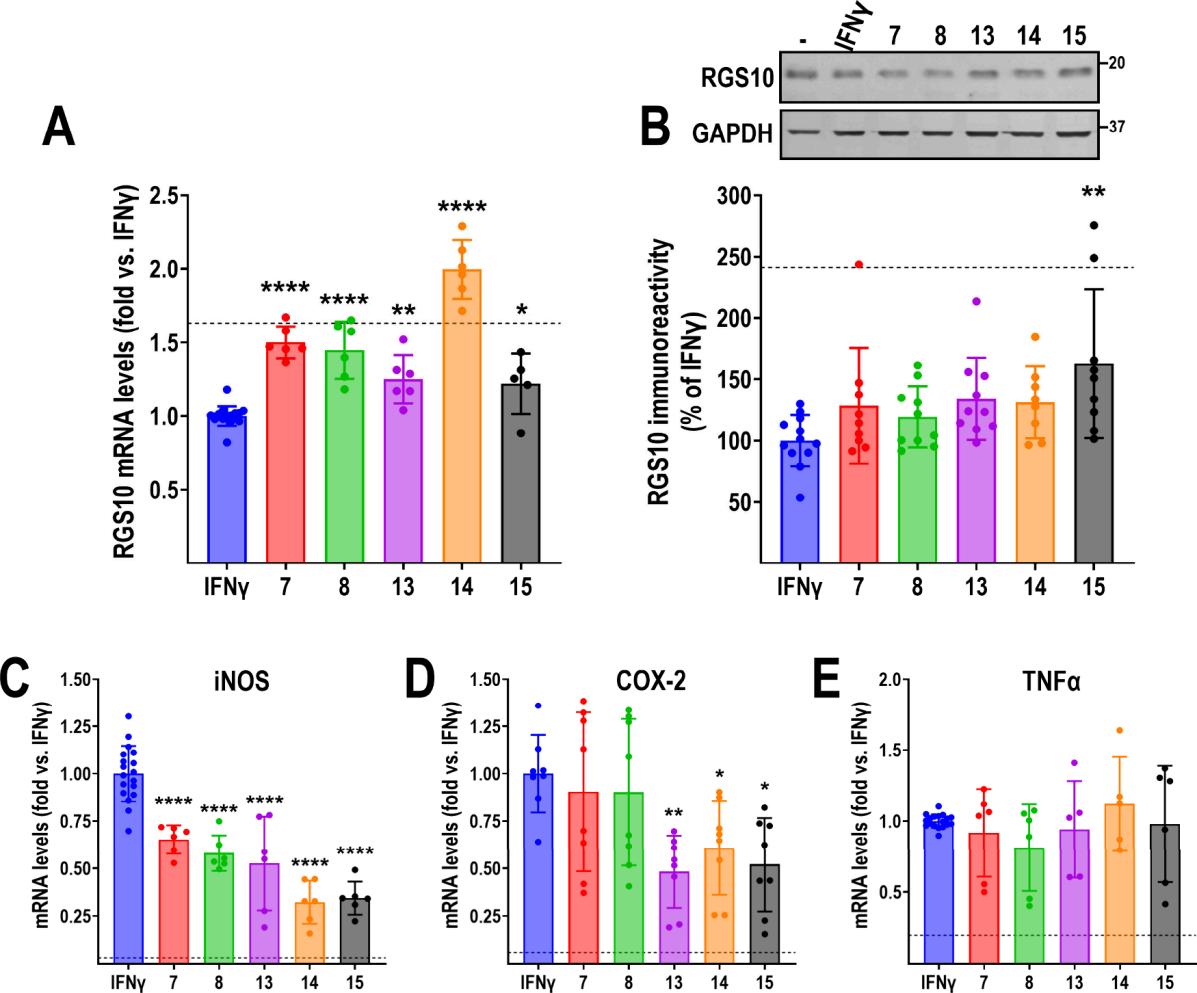
Effect of hits on RGS10 protein and mRNA, and inflammatory gene expression. All graphs are presented as levels normalized to those in the presence of IFNγ (10 ng/mL; 24 h); dashed line represents levels in the absence of stimulus. A. All five compounds significantly reverse IFNγ-induced RGS10 mRNA silencing. B. Representative blot and quantification of four independent experiments in duplicate showing the effects of compounds on RGS10 protein levels in BV-2 cells. While all compounds displayed a trend for reversing IFNγ-induced RGS10 suppression, only the effect of compound **15** reached significance. C. All five compounds significantly reverse the IFNγ-induced induction of iNOS mRNA. D. Compounds **13**, **14**, and **15** significantly reverse the IFNγ-induced induction of COX-2 mRNA. Compounds **7** and **8** have no effect. E. None of the compounds reverse IFNγ-induced induction of TNFα mRNA. Sequences for primers used for qRT-PCR are shown in Table S1. *P < 0.05; **P < 0.01; ****P < 0.0001 using one-way ANOVA with Dunnet’s post hoc test for pairwise comparisons.

To confirm that the effects of compounds on RGS10^HiBiT^ luminescence were not an artifact of our assay, we next validated our hits for their effect on native RGS10 protein and mRNA levels in BV-2 cells. All five compounds (**7**, **8**, **13**, **14**, and **15**; 20 μM) significantly reversed IFNγ-induced (10 ng/mL; 24 h) RGS10 suppression (Figure 6A). We next assayed compounds **7**, **8**, **13**, **14**, and **15** (at 20 μM) by Western blot for their ability to reverse IFNγ-induced (10 ng/mL) RGS10 suppression at 24 and 48 h. Neither compound had any effect at 24 h (data not shown). Further, while all hits displayed a trend toward reversing IFNγ-induced RGS10 protein levels at 48 h, only the effect of compound **15** reached statistical significance (Figure 6B).

### Compound Effects on Inflammatory Gene Expression

Our original hypothesis was that reversing IFNγ-induced RGS10 silencing in microglia would suppress inflammatory responses and lead to neuroprotection. Thus, we next assayed our top 5 compounds for their ability to reverse IFNγ-mediated induction of inflammatory gene expression. BV-2 cells were treated with IFNγ (10 ng/mL; 24 h) with or without compound (**7**, **8**, **13**, **14**, **15**; 20 μM). IFNγ robustly induced mRNA expression of the inflammatory genes iNOS, COX-2, and TNFα (Figures 6C–E). All five compounds significantly reversed the induction of iNOS expression; however, only compounds **14** and **15** reversed COX-2 mRNA expression. None of the compounds had any effect on the induction of TNFα mRNA. Altogether, these results indicate that in addition to their effect of RGS10 expression, they also affect inflammatory gene expression induced by IFNγ. Furthermore, the differential effects on iNOS, COX-2, and TNFα mRNA indicate distinct mechanism(s) of action for our validated hits, affecting separate targets within the IFNγ signaling axis.

### Compound **15** Reverses IFNγ-Induced RGS10 Silencing in a Concentration-Dependent Manner

**Figure 7:**
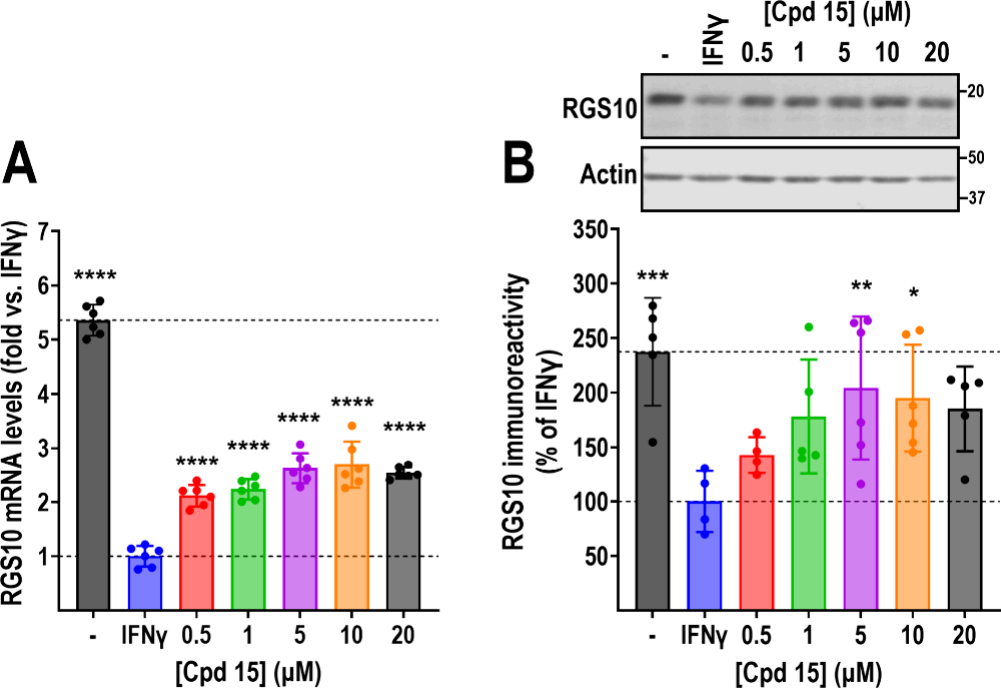
Compound 15 reverses IFNγ-induced silencing in a concentration-dependent manner. Compound **15** significantly reverses IFNγ-induced silencing of RGS10 mRNA (A) and protein (B), as measured by qRT-PCR and Western blot, respectively. Significant reversal occurs at concentrations as low as 0.5 μM for mRNA and 5 μM at the protein level. All graphs are presented as levels normalized to those in the presence of IFNγ (10 ng/mL; 24 h); top dashed line represents levels in the absence of stimulus. *P < 0.05; **P < 0.01; ***P < 0.001; ****P < 0.0001 using one-way ANOVA with Dunnet’s post hoc test for pairwise comparisons.

The screen and all follow-up studies were performed at a compound concentration of 20 μM, and our top five compounds all reversed IFNγ-induced RGS10 silencing with an EC_50_ value close to, or significantly above this concentration in the HiBit assay (Figure 5B). Because compound **15** was the only hit that significantly reversed IFNγ-induced RGS10 silencing at the protein level (Figure 6B) we assessed whether this effect would be maintained at lower concentrations. As shown in Figure 7, Compound **15** significantly reversed IFNγ-induced (10 ng/mL; 24 h) RGS10 silencing in a dose-dependent manner. RGS10 mRNA levels were significantly increased at all concentrations tested (0.5–20 μM; Figure 7A). While there was a trend for increased RGS10 protein levels at all concentrations, significant reversal was achieved only at concentrations at or above 5 μM (Figure 7B). The concentration of compound **15** needed to achieve significant reversal of IFNγ-induced RGS10 silencing in these experiments was significantly lower than the estimated EC_50_ value determined in the HiBit assay (Figure 5). However, the differences in assay setup, combined with the fact that the HTS assay is run in a single clone cell line, as opposed to the heterogeneous parental BV-2 population, may account for the differences in apparent potency.

## Discussion and Conclusions

In the current study, we explored the druggability of RGS10, a novel target to reduce pro-inflammatory microglial signaling that contributes to chronic neuroinflammation. Previous studies have identified reversal of RGS10 silencing as a promising therapeutic strategy for inflammatory NDs;^24−27,29^ however, until now, no chemical tools have been available to explore this. The hits identified in our screen represent the first examples of molecules with this ability. These tool compounds could be used to further elucidate the mechanisms by which RGS10 regulates microglial inflammatory functions in addition to serving as promising leads for future drug development efforts.

Since their discovery in the mid-1990s, multiple efforts have been made to target RGS proteins in drug discovery. While several inhibitors of the RGS–Gα interaction have been identified as active both in vitro^42^ and in vivo,^43^ enhancing the effect of an RGS protein remains far more challenging. We have shown that increasing RGS protein levels is sufficient to increase function.^44^ Thus, identifying mechanism(s) that regulate RGS protein expression is a valid strategy. Yet, for the majority of RGS proteins, these mechanisms are poorly understood. Our chosen screening strategy has the benefit of identifying RGS10-modulating compounds in a manner that is agnostic to the mechanism of action. Identification of the molecular target(s) of these compounds will unveil the regulatory machinery controlling RGS10 expression in microglia. In addition, to enable future drug development efforts, it will be important to determine the mechanism by which these compounds reverse IFNγ-induced RGS10 silencing.

Interestingly, none of the compounds identified here reverse LPS-induced silencing nor do they increase RGS10 levels in the absence of stimulus, indicating that the molecular target(s) for these compounds resides within the IFNγ signaling axis. Parallel regulation of the RGS10 protein and mRNA further indicates that these compounds act through a mechanism that impacts either transcriptional or epigenetic regulation of RGS10. RGS10 silencing in activated microglia requires histone deacetylation at the RGS10 promoter, and the broad-spectrum histone deacetylase (HDAC) inhibitor Trichostatin A (TSA) blocks RGS10 suppression by inflammatory stimuli in BV-2 cells and mouse primary microglia.^29^ Although HDAC inhibitors display anti-inflammatory effects in many systems,^45^ their use in chronic CNS disease is fundamentally problematic due to broad epigenetic effects and associated toxicities. Thus, it will be important to assess whether our currently identified hits and any future optimized compounds affect HDAC activity. However, the lack of efficacy in reversing LPS-induced RGS10 suppression (Table 1), which also requires the action of HDACs, indicates that our initial hits act through a mechanism distinct from HDAC inhibition.

Our future studies will include detailed elucidation of the molecular target for our identified hits, as well as chemical optimization to improve potency and efficacy. The differential effect observed on inflammatory gene expression (iNOS, COX-2, and TNFα) suggests that not all compounds act through the same mechanism. Compounds **7** and **8** only differ in the position of one fluorine (ortho- vs meta-) and show a similar pattern on inflammatory gene expression (suppressing iNOS, but not COX-2). Thus, these two compounds likely act on the same target. In contrast, compounds **13**, **14**, and **15** suppress both iNOS and COX-2 expression. While this may indicate a similar mechanism of action, the diverse structures of these three compounds could suggest the engagement of distinct molecular targets. Our future studies will utilize proteomic approaches to identify the target for these compounds. Until the target(s) for these compounds are identified, rational chemical optimization will be challenging.

In the current study, we opted to use the microglial cell line BV-2 due to the need to develop a reporter line amenable for HTS. This cell line has been well characterized to maintain many phenotypes associated with microglial function and where RGS10 is transcriptionally silenced by LPS. Here, we also show that IFNγ treatment will also result in RGS10 silencing, but with less associated toxicity than LPS (Figure 1). It will be important to confirm the effects of our identified hits in nonimmortalized cell systems, such as mouse primary microglia. In addition, there are known species differences in microglial behaviors, and as such, a human model will also need to be used to validate the effects of these, and any future leads, in relevant cell systems, as well as in vivo.

In addition to identifying the molecular target and performing chemical optimization to improve potency and efficacy of our hits, it will also be important to validate that reversing IFNγ-induced RGS10 silencing impacts not only inflammatory gene expression but also microglial properties associated with pro-inflammatory phenotypes. IFNγ promotes pro-inflammatory behaviors such as migration, which is closely linked to their neurotoxic effects in NDs. In order for an RGS10-modulating compound to be a valid lead for future drug development, it needs to reverse these pro-inflammatory behaviors as well. Previous studies using knock-down and overexpression of RGS10 in mouse and rat primary microglia suggest that this approach has the potential to be successful.^26,27^ While this will have to be experimentally validated, our identification of the first compounds with the ability to reverse IFNγ-induced RGS10 silencing represents an important first step.

## Materials and Methods

### Materials

All reagents were purchased from ThermoFisher Scientific (Waltham, MA), unless otherwise specified.

### Compound Purity Assessment

All compounds used in this study were purchased from reputable vendors, such as ChemDiv. ChemDiv provides 100% quality control for all compounds and guarantees at least 90% purity. QC data for compound **12** is shown in Figure S4. The top 5 compounds (confirmed actives) were confirmed in house to be >95% pure by HPLC analysis. Representative HPLC trace of compound **15** is shown in Figure S5. No in-house chemical synthesis was performed. Purity was determined by HPLC on an Agilent 1260 Infinity II Analytical HPLC using an Eclipse Plus C18 3.5 μm 4.6 × 100 mm column. Samples were diluted in 50/50 Acetonitrile:Water with 0.1% TFA. 75 μL was injected, and a gradient of 95% Water to 95% Acetonitrile over 30 min was used. Purity was determined by the area under the peak in both channels using the Agilent software.

### Cell Culture

BV-2 murine microglial cells were kindly donated by Dr. Shelley Hooks (University of Georgia). BV-2 cells were cultured in Dulbecco’s modified Eagle’s medium (DMEM; no. 11995065) supplemented with 10% fetal bovine serum (FBS; no. 16000044) and 1% antibiotic-antimycotic (100X; no. #15240062). Cells were maintained at 37 °C, with 5% CO_2_ content and standard humidity.

### Preparation of Cell Lysates

Cells were harvested on ice in lysis buffer containing protease inhibitors (20 mM Tris-HCl (pH 7.4), 150 mM NaCl, 1 mM EDTA, 1 mM β-glycerophosphate, 1% Triton X-100, 0.1% SDS, and cOmplete Protease Inhibitor Cocktail EDTA-free (Roche, #11836170001)). Lysates were sonicated for 10 min at 4 °C and centrifuged at 6000 rpm for 3 min. The supernatants were used for SDS-PAGE and Western blot. Protein concentration was determined using the Pierce BCA Protein Assay (#23225). Protein concentration was adjusted in each sample to allow for equal protein loading, and sample buffer (Li-Cor; Lincoln, NE; #928-40004) was added before loading samples on the gel.

### SDS-PAGE and Western Blot

Equal amounts of proteins were loaded onto a 12% SDS/PAGE gel and resolved at 160 V and 0.4 A for 1 h. Proteins were transferred to an Immobilon-P PDVF membrane (EMD Millipore, Burlington, MA; #IPVH00010) for 2 h at 160 V, 0.4 A. Following protein transfer, the membrane was blocked at room temperature for 1 h with Intercept PBS Blocking buffer (Li-Cor, #927-70001) and then incubated in primary antibodies (goat anti-RGS10 (1:1,000; #sc-6206, Santa Cruz Biotechnologies, Santa Cruz, CA), rabbit anti-β-Actin (1;5,000; #A2066, Sigma-Aldrich, St. Louis, MO), and rabbit anti-GAPDH (1:1,000, #5174S, Cell Signaling, Danvers, MA) for 2 h at room temperature. Primary antibodies were diluted in Intercept T20 Antibody PBS diluent (Li-Cor, #927-75001). The membrane was subsequently incubated for 1 h in donkey antigoat IRDye 800CW (1:25,000; Li-Cor; #926-32214), and goat antirabbit IRDye 680RD (1:15,000; Li-Cor; #926-68071) secondary antibodies. Following each antibody incubation, membranes were washed four times with PBS and 0.1% Tween-20. Membranes were imaged by using the Azure600 imaging system (Azure Biosystems, Dublin, CA).

### qRT-PCR

RNA was isolated from BV-2 cells using the RNeasy Mini Kit (no. 74104; Qiagen, Germantown, MD). Isolated RNA was quantified using the ThermoFisher NanoDrop One spectrophotometer. Reactions for the qRT-PCR were set up using the Luna Universal One-Step RT-qPCR kit (#E3005; New England Biolabs, Ipswich, MA). Briefly, in a 96-well plate, 20 μL reaction mixtures were prepared containing 100 ng of template RNA, 0.4 μM primers (for primer sequences, see Table S1), Luna Universal One-Step Reaction Mix and Luna WarmStart RT Enzyme Mix. RT-PCR was run using the QuantStudio 3 system (Applied Biosystems, Carlsbad, CA). Briefly, RNA was reverse transcribed at 55 °C for 10 min, followed by initial denaturation at 95 °C for 1 min. 40 cycles of denaturation (95 °C for 10 s) and extension (60 °C for 1 min) were completed. At the end of each cycle, the plate was read to obtain the Cq values. Finally, a melt curve was generated following the instrument’s melt curve protocol where the samples were subjected to the following cycle −95 °C for 15 s, 60 °C for 1 min, and 95 °C for 15 s.

### CRISPR-Cas9 Design

Alt-R CRISPR-Cas9 crRNA (AGCTTATGTGTTGTAAATTC) targeting the 3′ end of the *RGS10* locus was purchased from Integrated DNA Technologies (IDT; San Diego, CA). Alt-R CRISPR-Cas9 tracrRNA–ATTO 550 (no. 1075927) and Alt-R S.p. Cas9 Nuclease V3 (#1081058) were purchased from IDT. Design for HiBiT donor template (ssODN) included the DNA sequence for the HiBiT tag flanked by sequences homologous to the region directly upstream and downstream of the mouse *RGS10* 3′ end. The ssODN sequence was as follows (HiBiT sequence underlined):

GGAAGAAGAGCCCCCGGATGCTCAGACCGCAGCTAAGC-GAGCCTCCAGAATTTACAACACAGTGAGCGGCTGGCGG-CTGTTCAAGAAGATTAGCTAAGCTGAGCCCTTCACCCC-AGCGAAGGAGAGGGAT

### Development of a BV-2-RGS10^HiBiT^ Stable Cell Line

Low passage BV2 cells were seeded into a 10 cm culture plate and allowed to reach 70–85% confluency, indicative of an active growth phase. 20 μL of 100 μM crRNA (in TE) and 20 μL of 100 μM tracrRNA were mixed to achieve a final concentration of 50 uM gRNA duplex. This gRNA duplex was heated in a PCR block at 95 °C for 5 min and then allowed to cool to room temperature. For RNP complex formation, 26.22 μL of 61 μM Cas9 Nuclease V3 was mixed with 38.40 μL of 50 μM gRNA duplex (1:1.2 molar ratio, respectively) to a final volume of 80 μL using sterile PBS and incubated at room temperature for 10–20 min. Cells were trypsinized, and 4 million cells were collected, pelleted by centrifugation, and resuspended in 5 mL of PBS. Cells were pelleted again and resuspended in 300 μL of Opti-MEM (#31985070). A 300 μL portion of the cell suspension was mixed with 80 μL of the RNP complex and 20 μL of 4 μM donor template (HiBiT ssODN) in a chilled sterile cuvette. This mixture was then subjected to electroporation using a Bio-Rad Gene Pulser Xcell Eukaryotic System (Hercules, CA) with the settings of 260 V and 975 μF with a decay wave. Immediately following this, cells were transferred to a 25 cm^2^ flask with complete media and allowed to recover for 24 h.

Twenty-four hours after electroporation, cells were resuspended in 1 mL of PBS with 0.1% BSA. Cells were sorted using the 550 nm laser of the BD Biosciences FACS Aria III cell sorter (Franklin Lakes, NJ), utilizing the fluorescent tracrRNA–ATTO 550 to identify positive cells. Of the ATTO 550-positive cells, the top 30% with the strongest signal were used to sort one cell per well of multiple 96-well plates. Single clone colonies were allowed to form before expansion to 6-well plates. Once individual wells reached confluency, clones were tested for luminescence signal (baseline and in the presence of 10 ng/mL IFNγ) using the Nano-Glo HiBiT Lytic Detection System (described below).

### Cell Plating and Treatments

BV-2-RGS10^HiBiT^ cells were seeded into a 150 cm^2^ flask and maintained in assay media (DMEM without phenol red (#21063029), 10% ultralow IgG FBS (#A3381901)). At 80–90% confluency, cells were trypsinized and resuspended in assay media. 30 μL of cell suspension was dispensed into each well of 384-well CulturPlate (#6007680; PerkinElmer, Waltham, MA). Plates were centrifuged for 1 min at 1,000 rpm. IFNγ (#485-MI-100; R&D systems, Minneapolis, MN) or LPS (#L2880; Sigma-Aldrich) was diluted in assay media to a concentration of 40 ng/mL, and 10 μL was dispensed into wells for a final concentration of 10 ng/mL. In negative control wells, 10 μL of assay media was dispensed into wells. Plates were centrifuged for 1 min at 1,000 rpm, incubated at room temperature for 1 h, and placed in an incubator at 37 °C until assayed.

### Cell Viability Assay

Glycylphenylalanyl-aminofluoro-coumarin (GF-AFC; #03AFC033-CF, MP BioMedicals, Irvine, CA) stock (75 mM) was diluted 1:2,000 in 100 mM HEPES. Media was removed from the microplate using an ELx405 CW plate washer (BioTek, Winooski, VT), and 20 μL of GF-AFC was added. The plate was centrifuged for 1 min at 1,000 rpm and incubated at 37 °C for 30 min before reading fluorescence (390_EX_/505_EM_) on a Synergy Neo2 multimode plate reader (BioTek).

### Nano-Glo HiBiT Lytic Detection Assay

Components of the Nano-Glo HiBiT Lytic Detection System (no. N3030; Promega, Madison, WI) were prepared following the manufacturer protocol. The lytic buffer was warmed to 37 °C before use, and then LgBiT protein (1:100) and Nano-Glo HiBiT lytic substrate (1:50) were added. Reagent mixture was mixed gently on a rotator for 30 min before use, then 20 μL was added into each well. The plate was centrifuged for 1 min at 1,000 rpm, incubated on an orbital shaker for 7 min at 600 rpm, and incubated for 15 min at room temperature. Luminescence was detected on the Synergy Neo2 multimode plate reader using a 1.0 s integration time and 8.0 mm read height.

### Small Molecule Screening Library

The CNS BBB library available from ChemDiv (San Diego, CA) contains 23,432 compounds; the first 9,600 compounds were screened here. Detailed library and compound information available at: https://www.chemdiv.com/catalog/focused-and-targeted-libraries/cns-bbb-library. This collection is preselected using parameters favorable for blood–brain-barrier (BBB) penetration, and other properties making them favorable candidates for CNS action.^37,38^ The library and compounds used for follow-up studies were purchased from ChemDiv and validated for purity as described under Materials.

### Primary Screen

The screen was performed at the Purdue University Chemical Genomics Facility (CGF). Compounds were screened in the presence of IFNγ (10 ng/mL, 48 h) to identify hits that would reverse IFNγ-induced RGS10 silencing. Cells were plated at a density of 2,500 cells/well, as described above, and 80 nL compound (10 mM) was added directly following IFNγ using a Beckman Coulter Echo 525 acoustic liquid handler (Brea, CA), to a final concentration of 20 μM. Following 48 h incubation at 37 °C, cell viability and HiBiT luminescence was determined as described above. Hits were defined as compounds that increased normalized luminescence (RLU/RFU) > 2 SD above that of IFNγ alone.

### Ligand Clustering Analysis

Ligand clustering was performed using Schrodinger’s molecular modeling. The radial-type fingerprint approach was applied with the fingerprint set to 64-bit precision. To generate these fingerprints, an atom-typing scheme was applied that categorizes atoms by functional type: hydrogen (H), carbon (C), halogen grouped as [fluorine, chlorine (F, Cl)] and [bromine, iodine (Br, I)], pnictogens and chalcogens grouped as [nitrogen, oxygen (N, O)] and [sulfur (S)], with all other atom types categorized as “others”. Bonds were differentiated by their hybridization states. A Tanimoto coefficient-based similarity matrix was utilized for the comparative analysis, employing CGF-0194281 as a reference compound for the assessment of the structural similarity among the analyzed ligands. The Tanimoto similarity index was calculated based on the following formula: c/(a+b–c). [c = Number of bits that are on in both structure 1 and structure 2, a = Number of bits that are on in structure 1, b = Number of bits that are on in structure 2].^39^ Separation ratio across different numbers of clusters, distance matrix, and dendrogram illustrating the arrangement of the clusters are presented in Figures S1–S3. The full cluster analysis, with Tanimoto scores, is presented in Table S2.

### Data Analysis

Western blot images were quantified by using Image Studio software (Li-Cor Biosciences). The intensities of bands for RGS10 were normalized to Actin or GAPDH as a loading control. qRT-PCR data was analyzed using the Cq and ΔΔCq values were determined using the ThermoFisher Design and Analysis application. All data were analyzed using GraphPad Prism 10 (GraphPad, La Jolla, CA). Dose–response curves were fit using nonlinear regression. Data sets with three or more groups were analyzed with one-way ANOVA, with Dunnet’s post hoc test for multiple comparisons. All experiments were run at least three times. Data are presented as mean ± SD with a *P*-value less than 0.05 considered significant.

## Supporting Information Available

Primers used for qRT-PCR (Table S1), Cluster analysis of confirmed hits (Table S2), Separation ratio across different numbers of clusters (Figure S1), Distance matrix (Figure S2), Dendrogram illustrating the arrangement of the clusters (Figure S3), Representative purity determination (assessed by vendor) for compounds reordered for confirmation (Figure S4), Representative HPLC trace for compound **15** (Figure S5) (PDF)
Molecular Formula Strings (CSV)

## Abbreviations

AD: Alzheimer’s Disease
COX-2: cyclooxygenase 2
GAP: GTPase activating protein
GPCR: G protein-coupled receptor
IFNγ: Interferon-γ
iNOS: inducible nitric oxide synthase
LPS: Lipopolysaccharide
ND: Neurodegenerative Disease
PD: Parkinson’s Disease
RGS: Regulator of G protein signaling
TLR: Toll-like receptor
TNFα: tumor necrosis factor α
