# Supplementary material for: A Phenotypic High-Throughput Screen Identifies Small Molecule Modulators of Endogenous RGS10 in BV-2 Cells
Source: J Med Chem. 2024 Nov 15;67(22):20343–52. doi: 10.1021/acs.jmedchem.4c01738 (PMC11613444; doi:10.1021/acs.jmedchem.4c01738)
Supplement: Supplementary file 1 — jm4c01738_si_001.pdf [file jm4c01738_si_001.pdf]

SUPPORTING INFORMATION

A phenotypic high-throughput screen identifies small molecule modulators of endogenous RGS10 in BV-2 cells.

Shwetal Talele[1], Stephanie Gonzalez[2], Julia Trudeau[1], Ahmad Junaid[2], Cody A. Loy[1], Ryan A. Altman[2] and Benita Sjögren[1,2]*

[1]Department of Pharmaceutical Sciences, University of California, Irvine, Irvine, CA 92697

[2] Borch Department of Medicinal Chemistry and Molecular Pharmacology, Purdue University, West Lafayette, IN 47907

Correspondence: jsjogren@uci.edu

TABLE OF CONTENTS

Table S1. Primers used for qRT-PCR. S2

Table S2. Cluster analysis of confirmed hits. S3

Figure S1. Separation ratio across different numbers of clusters. S7

Figure S2. Distance matrix. S7

Figure S3. Dendrogram illustrating the arrangement of the clusters. S8

Figure S4. Representative purity determination for compounds re-ordered for confirmation. S9

Figure S5. Representative HPLC trace for compound 15 S10

Table S1. Primers used for qRT-PCR.

GENE	FORWARD PRIMER	REVERSE PRIMER
GAPDH	5’-TGG CCT TCC GTG TTC CTA C-3’	5’-GAG TTG CTG TTG AAG TCG CA-3’
RGS10	5’- GGA GAA TCT TCT GGA AGA CC-3’	5’-CTG CTT CCT GTC CTC CGT TTT C-3’
INOS	5’-CAG CTG GGC TGT ACA AAC CTT-3’	5’-CAT TGG AAG TGA AGC GGT TCG-3’
TNFA	5’-CCT GTA GCC CAC GTC GTA C-3’	5’-GGG AGT AGA CAA GGT ACA ACC C-3’
COX-2	5’-TGC AAG ATC CAC A**G**C CTA CC-3’	5’-GCT CAG TTG AAC GCC TTT TG-3’

Table S2. Cluster analysis of confirmed hits. Compounds confirmed to significantly reverse IFNγ-induced RGS10 silencing where subjected to chemical clustering as described in *Materials & Methods*.

Cluster No.	Molecule Name	ChemDiv ID Number	Molecular weight (g/mol)	Structure	Tanimoto Similarity
1	CGF-0194281	L923-0739	427.33	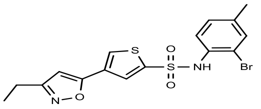	1
2	CGF-0188681	S348-2010	470.452	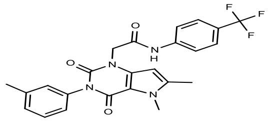	0.07
2	CGF-0188926	S348-1665	499.34	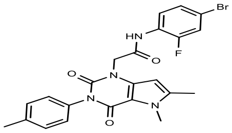	0.098
3	CGF-0185364	P194-2174	387.443	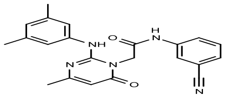	0.073
4	CGF-0190170	S396-0761	334.42	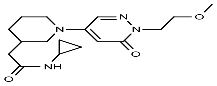	0.048
5	CGF-0187661	S296-4338	382.391	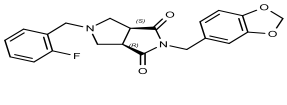	0.064
6	CGF-0189561	S368-0654	381.363	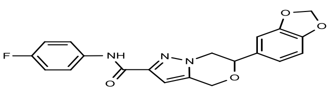	0.065
7	CGF-0190549	S425-0152	336.388	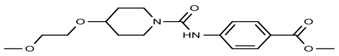	0.052
8	CGF-0185111	M788-4605	399.447	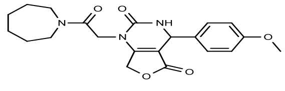	0.057
8	CGF-0186074	P803-0970	419.91	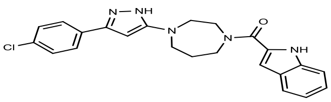	0.075
8	CGF-0186204	P805-0787	419.91	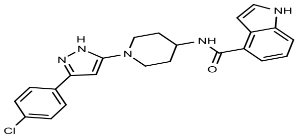	0.084

Table S1 cont.

Cluster No.	Molecule Name	ChemDiv ID Number	Molecular weight (g/mol)	Structure	Tanimoto Similarity
8	CGF-0186224	P809-0783	405.89	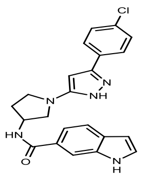	0.082
8	CGF-0186250	P809-0586	371.444	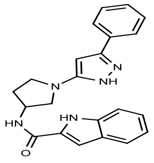	0.083
8	CGF-0186306	P809-0837	445.75	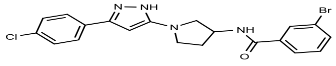	0.101
8	CGF-0186316	P809-0986	377.47	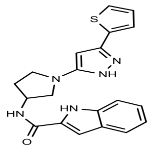	0.085
8	CGF-0186330	P809-0588	388.515	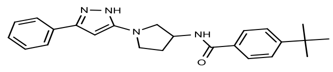	0.095
8	CGF-0188707	S350-0115	433.8	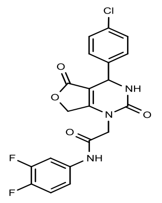	0.046
8	CGF-0188747	S350-0116	433.8	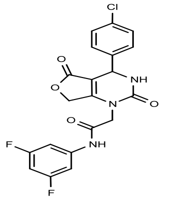	0.047
8	CGF-0188861	S350-0070	476.71	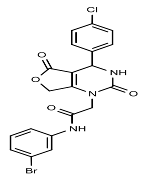	0.059
8	CGF-0188910	S350-0562	470.323	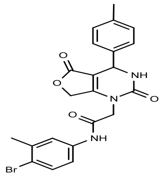	0.095

Table S1 cont.

Cluster No.	Molecule Name	ChemDiv ID Number	Molecular weight (g/mol)	Structure	Tanimoto Similarity
8	CGF-0188947	S350-0139	476.31	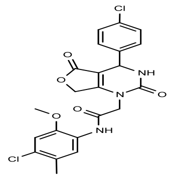	0.064
8	CGF-0189863	S380-0696	414.506	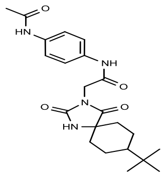	0.05
8	CGF-0185111	C522-3730	496.99	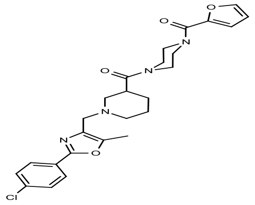	0.055
9	CGF-0193870	F326-0563	372.468	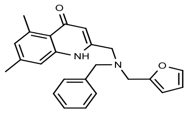	0.078
10	CGF-0187430	S324-0329	374.48	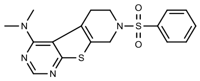	0.07
10	CGF-0187586	S324-0192	363.48	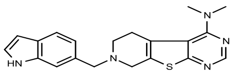	0.045
10	CGF-0187624	S324-0170	313.42	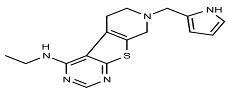	0.071
10	CGF-0187664	S324-0173	325.43	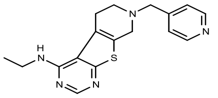	0.061
11	CGF-0193160	8013-2958	288.342	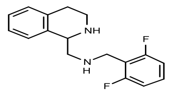	0.038
12	CGF-0193338	C598-0583	380.87	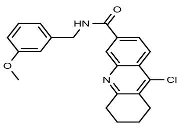	0.056
13	CGF-0185946	P759-1154	340.427	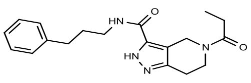	0.069

Table S1 cont.

Cluster No.	Molecule Name	ChemDiv ID Number	Molecular weight (g/mol)	Structure	Tanimoto Similarity
14	CGF-0185944	P742-2340	353.422	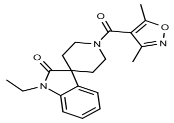	0.077
15	CGF-0194077	G332-0404	417.59	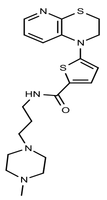	0.056
16	CGF-0193569	E722-1455	448.04	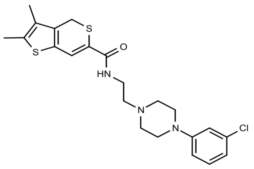	0.063
17	CGF-0188140	S342-0449	380.35	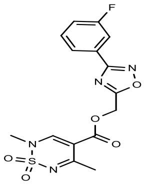	0.088
18	CGF-0188561	S343-0670	363.417	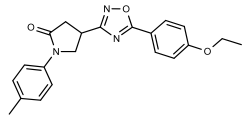	0.097
19	CGF-0189560	S360-0441	321.296	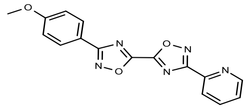	0.07

SUPPLEMENTAL FIGURES

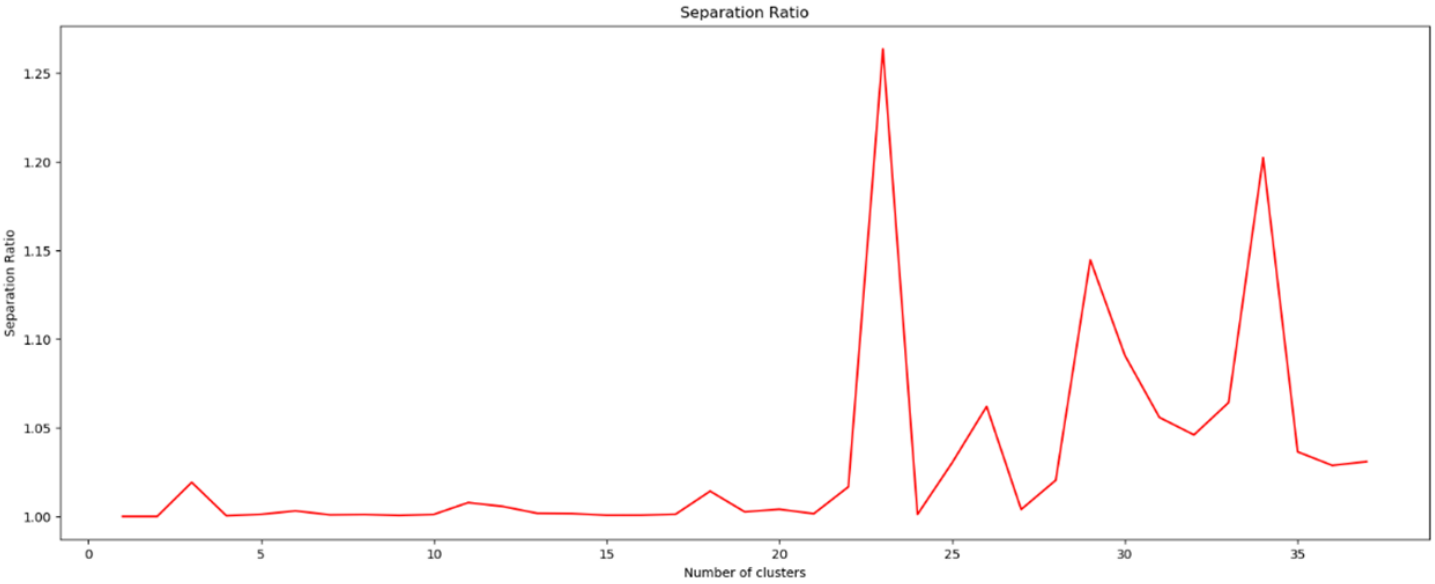

Figure S1. Separation ratio across different numbers of clusters. The general distinctiveness of clusters remains stable across various cluster counts. However, there are specific points where the distinctiveness significantly improves.

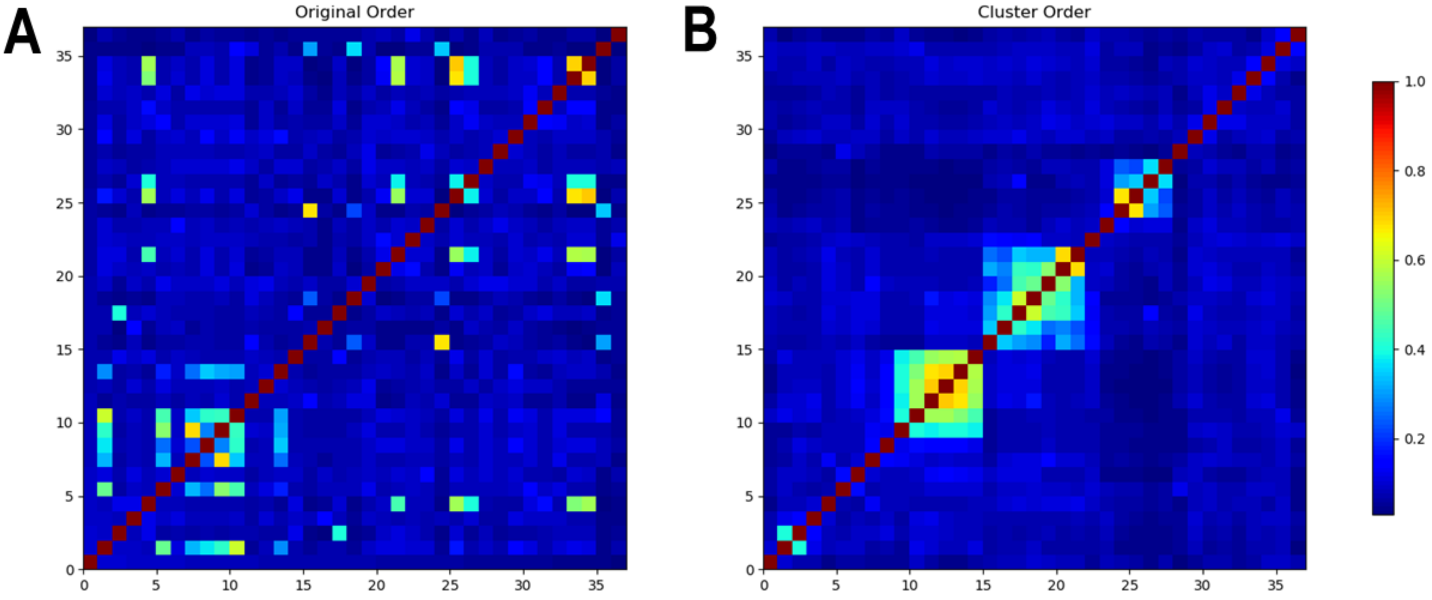

Figure S2. Distance matrix. A. The original order of the data points does not reveal any obvious clustering patterns. The distances between points are scattered without a discernible structure, suggesting that the data points are not arranged in any meaningful order. **B. Cluster order;** After reordering the data points based on clustering results, distinct blocks of higher similarity (red and yellow) become apparent. These blocks along the diagonal indicate that the clustering algorithm successfully grouped similar data points together, making the clusters more visible and distinct.

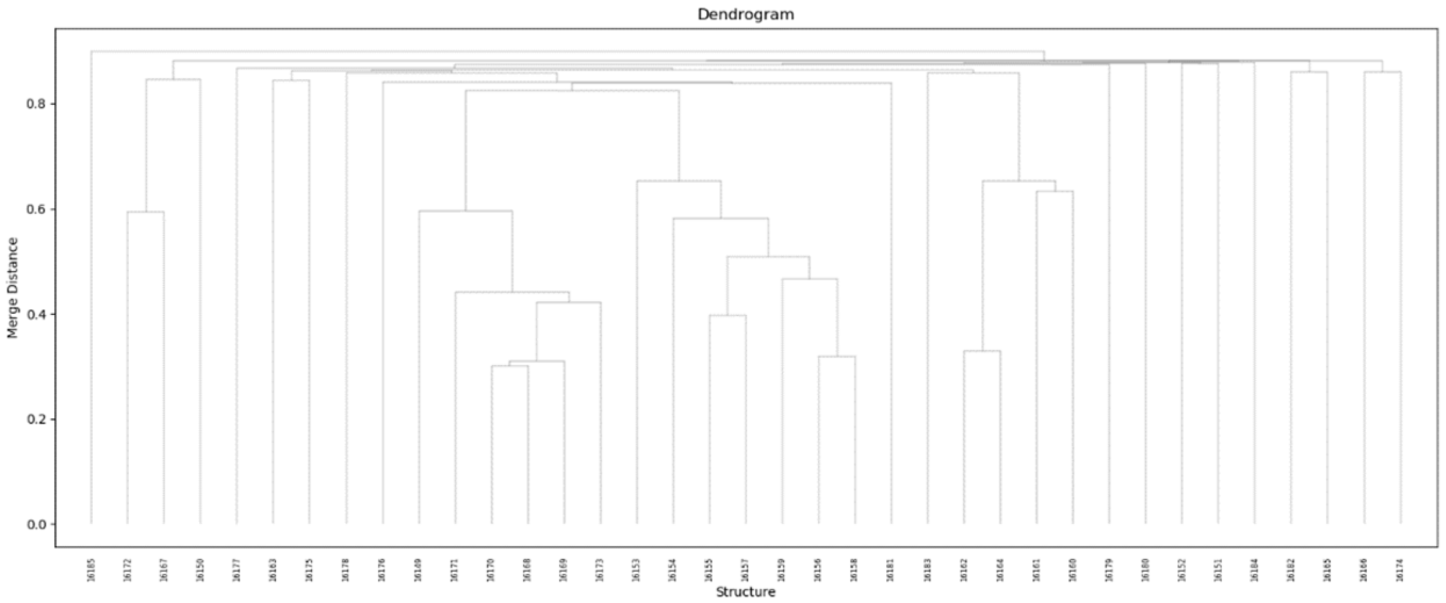

Figure S3. Dendrogram illustrating the arrangement of the clusters. The X-axis represents the individual data points or structures being clustered. Each label corresponds to a specific data point in the dataset. The Y-axis represents the merge distance, which is a measure of dissimilarity between clusters. The higher the merge distance, the more dissimilar the clusters being joined. The dendrogram shows distinct clustering patterns, with some clusters being formed at very low merge distances, indicating high similarity among those data points.

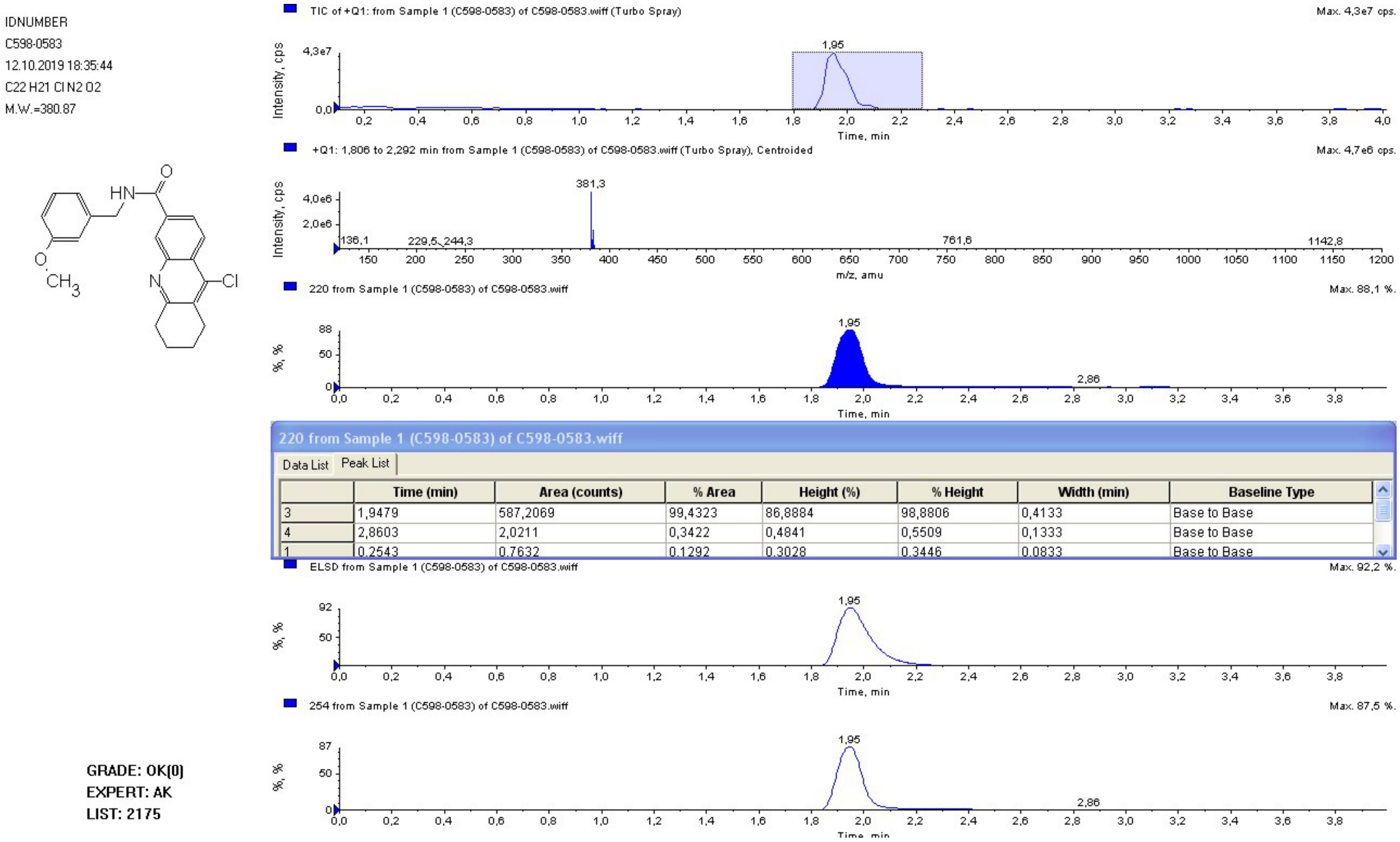

	Time (min)	Area (counts)	% Area	Height (%)	% Height	Width (min)	Baseline Type
3	1,9479	587,2069	99,4323	86,8884	98,8806	0,4133	Base to Base
4	2,8603	2,0211	0,3422	0,4841	0,5509	0,1333	Base to Base
1	0,2543	0,7632	0,1292	0,3028	0,3446	0,0833	Base to Base

Figure S4. Representative purity determination for compounds re-ordered for confirmation. QC data supplied by vendor (ChemDiv). The purity accuracy is confirmed by 1H NMR and LC (UV)/MS spectra for all re-ordered compounds. The purity was determined to be >90% by these methods.

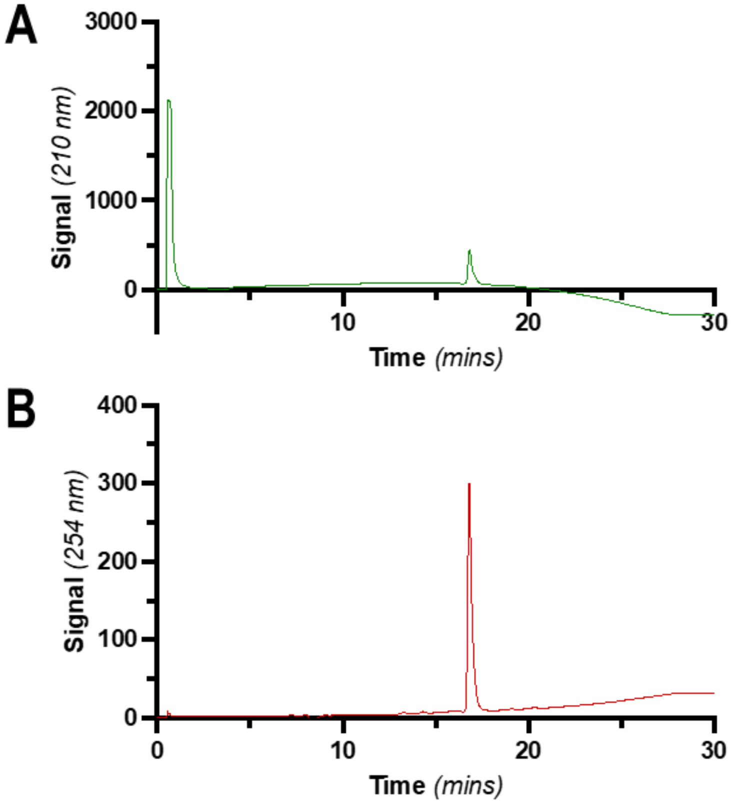

Figure S5. Representative HPLC trace for compound 15. A. 210 nm channel (green) **B.** 254 nm channel (red). Compound was determined to be > 95% pure based on peak traces in both channels.
